# A systematic review on malaria sero-epidemiology studies in the Brazilian Amazon: insights into immunological markers for exposure and protection

**DOI:** 10.1186/s12936-017-1762-7

**Published:** 2017-03-07

**Authors:** Pedro M. Folegatti, André M. Siqueira, Wuelton M. Monteiro, Marcus Vinícius G. Lacerda, Chris J. Drakeley, Érika M. Braga

**Affiliations:** 1 Department of Immunology and Infection, London School of Hygiene & Tropical Medicine, Keppel Street, London, WC1E 7HT UK; 2 Instituto Nacional de Infectologia Evandro Chagas, Fundação Oswaldo Cruz, Rio de Janeiro, RJ Brazil; 3 Fundação de Medicina Tropical Dr. Heitor Vieira Dourado, Manaus, Brazil; 4 Escola Superior de Ciências da Saúde, Universidade do Estado do Amazonas, Manaus, Amazonas Brazil; 5 Instituto de Pesquisas Leônidas e Maria Deane, Manaus, Amazonas Brazil; 6 Departamento de Parasitologia, Universidade Federal de Minas Gerais, Av. Antônio Carlos 6627, Pampulha, Belo Horizonte, MG 31270-901 Brazil

**Keywords:** *Plasmodium falciparum*, *Plasmodium vivax*, Antibodies, Brazil, Sero-epidemiology, Systematic review

## Abstract

**Background:**

Considerable success in reducing malaria incidence and mortality has been achieved in Brazil, leading to discussions over the possibility of moving towards elimination. However, more than reporting and counting clinical cases, elimination will require the use of efficient tools and strategies for measuring transmission dynamics and detecting the infectious reservoir as the primary indicators of interest for surveillance and evaluation. Because acquisition and maintenance of anti-malarial antibodies depend on parasite exposure, seroprevalence rates could be used as a reliable tool for assessing malaria endemicity and an adjunct measure for monitoring transmission in a rapid and cost-effective manner.

**Methods:**

This systematic review synthesizes the existing literature on seroprevalence of malaria in the Brazilian Amazon Basin. Different study designs (cross-sectional surveys and longitudinal studies) with reported serological results in well-defined Brazilian populations were considered. Medline (via PubMed), EMBASE and LILACS databases were screened and the articles were included per established selection criteria. Data extraction was performed by two authors and a modified critical appraisal tool was applied to assess the quality and completeness of cross-sectional studies regarding defined variables of interest.

**Results:**

From 220 single records identified, 23 studies were included in this systematic review for the qualitative synthesis. Five studies reported serology results on *Plasmodium falciparum*, 14 papers assessed *Plasmodium vivax* and four articles reported results on both *Plasmodium* species. Considerable heterogeneity among the evaluated malarial antigens, including sporozoite and blood stage antigens, was observed. The majority of recent studies analysed IgG responses against *P. vivax* antigens reflecting the species distribution pattern in Brazil over the last decades. Most of the published papers were cross-sectional surveys (73.9%) and only six cohort studies were included in this review. Three studies pointed to an association between antibodies against circumsporozoite protein of both *P. falciparum* and *P. vivax* and malaria exposure. Furthermore, five out 13 cross-sectional studies evidenced a positive association between IgG antibodies to the conserved 19-kDa C-terminal region of the merozoite surface protein 1 of *P. vivax* (PvMSP1_19_) and malaria exposure.

**Conclusions:**

This systematic review identifies potential biomarkers of *P. falciparum* and *P. vivax* exposure in areas with variable and unstable malaria transmission in Brazil. However, this study highlights the need for standardization of further studies to provide an ideal monitoring tool to evaluate trends in malaria transmission and the effectiveness of malaria intervention programmes in Brazil. Moreover, the score-based weighted tool developed and used in this study still requires further validation.

## Background

Recent emphasis and constant efforts on malaria elimination and eradication goals are changing the global malaria epidemiology scenario. Indeed, there has been an extraordinary scale-up of malaria control interventions over the last decade, which resulted in a substantial decline in global malaria morbidity and mortality [1]. In this perspective, the Americas have made substantial progress in reducing malaria cases. Indeed, 18 of the 21 countries in the region are now classified as being in the pre-elimination and elimination phases. On the other hand, four countries accounted for 83% of cases reported in the Americas in 2015: Venezuela (30%), Brazil (24%), Peru (19%), and Colombia (10%) [1]. Despite the considerable number of cases which still requires attention (143,000 cases in 2015), Brazil achieved a significant decrease in malaria incidence over the last 15 years [2]. Nowadays, an optimistic approach highlights mainly *Plasmodium falciparum* elimination targets, the species responsible for <15% of all malaria cases diagnosed in the Brazilian Amazon Basin.

Because past and current malaria control policies and implementation strategies have successfully reduced transmission in Brazil, the measurement of malaria-associated morbidity may become increasingly difficult which may undermine elimination efforts. Moreover, it is worth noting that surveillance methods based solely on detection of symptomatic clinical illness are unlikely to provide accurate estimates of ongoing transmission in the Brazilian Amazon Basin [2–4]. Under these circumstances, monitoring malaria transmission is pivotal for estimating disease burden, planning control strategies and evaluating the impact of malaria control interventions. Indeed, surveillance systems can help programme managers to reduce malaria transmission by providing information on the populations in which incidence of malaria is highest and, therefore, to whom resources should be targeted. They also provide information on changes in incidence over time that require attention [5, 6]. However, there is no consensus about which methods are more refined and adequate to monitor changes in transmission intensity to support malaria control programmes.

Malaria infection imposes an antibody ‘footprint’ that will last longer than the infection itself. Anti-malarial antibody responses reflect cumulative malaria exposure in a population and may be considered an alternative tool to measure malaria transmission intensity as well as to evaluate changes in exposure [7]. Previous studies have shown that serological prevalence with age-stratified sampling allowing for calculation of seroconversion rates is an important adjunct measure for monitoring transmission and to provide key information for control programmes on malaria transmission patterns, especially when parasite rates are low [8–10]. In contrast to the lifespan of the vector or the half-life of discrete infections, antibodies persist markedly longer than individual malaria infections or infected mosquitoes, which suggests that seroprevalence rates could provide a reliable tool for assessing malaria endemicity [11].

There have been several studies investigating antibody responses against different malaria antigens in Brazil. However, there is a great dissimilarity between them, which may prevent a conclusive analysis to establish a comprehensive panorama of malaria epidemiology over the past decades in Brazil. Therefore, the overall aim of this study is to collate data from relevant serological surveys of anti-malarial antibodies conducted in Brazil through a systematic review strategy. Specific objectives of this study include: (a) the description of representative studies on seroprevalence of antibodies against *P. vivax* and *P. falciparum* in Brazil; (b) list antigenic-used targets according to their usability; and, (c) identify knowledge gaps for malaria serological research in Brazil.

## Methods

### Search strategy

The search comprised different studies designs (cross-sectional surveys and longitudinal studies) with reported serological results (seropositivity proportions and antibody levels) in well-defined populations. Papers included in this review were searched using Medline (via PubMed), EMBASE and LILACS (Latin America and Caribbean Health Sciences Literature) databases published up until 25 May 2016. The search was conducted using the following search terms: ‘Malaria AND (vivax OR *falciparum*) AND (serology OR antibody OR antibody OR immunoglobulin) AND Brazil’.

### Inclusion criteria

This systematic literature review included studies reporting data from serological surveys of antibodies against malarial antigenic targets (whole parasites, recombinant and synthetic peptides) in addition to the following inclusion criteria: (1) studies must have provided a description of markers and laboratory methods used for the surveys; (2) data on the population age structure and geographical location characteristics must have been reported; and, (3) description of sample strategy and/or study design should have been reported. Studies that measured total IgG, total IgM or IgG sub-classes humoral immune responses were considered. This review included articles written in both English and Portuguese.

### Exclusion criteria

This review excluded case reports, experimental studies in animal models, studies based solely on blood bank volunteers, pregnant women, and any other particular groups that were not considered to be representative of the general population of interest. Studies were also excluded if the recruitment of individuals was based on their clinical status (all malaria-positive individuals) or if only IgG sub-classes (IgG_1_, IgG_2_, IgG_3_, IgG_4_) were analysed. Reports with insufficient data, literature reviews and studies from non-endemic areas (imported cases or outbreaks outside the Brazilian Amazon endemic area) have also been excluded.

### Data extraction

Two independent review authors used the inclusion and exclusion criteria to identify possible studies based on titles and abstracts. Relevant information from the selected articles was extracted and entered into a standardized Excel datasheet by the same two authors independently. Any differences were resolved on further discussion with a third author before including the data in the review. Data were extracted using a pre-defined form, which included the following information: publication data (journal, authors, period of study, year of publication); demographic and ecological characteristics of the study site (rural *vs* urban, transmission variation due to seasonality); study design and sampling procedures, serological markers and methods used for measurement; parasite diagnostic methods used [microscopy or polymerase chain reaction (PCR)]; study population characteristics (age distribution and gender); seroprevalence (overall and antigen specific); parasite prevalence and transmission intensity in the study site (annual parasite index or yearly incidence).

### Critical appraisal

Most of the validated tools or checklists to appraise the quality of cross-sectional studies have been designed to assess randomized interventional studies. In 1998, Downs and Black [12] proposed a checklist to assess the methodological quality of randomized and non-randomized studies of health care interventions, which has been used in a number of systematic reviews since then. The Newcastle-Ottawa Scale (NOS) was developed to assess the quality of non-randomized studies (case–control and cohort) and has also been used recently as a quality assessment tool in systematic reviews and meta-analysis [13]. Despite these two existing checklists for non-randomized studies, none of them was designed to assess cross-sectional research as they mainly focus on cohort and case–control studies. A new weighted tool was developed based on these modified checklists proposed by Downs and Black and the NOS (see Table 1). Articles were assessed against a score-based system, which combined elements of both these scales, giving weight to studies whose samples were truly representative of the population. Papers with a score >70% were included in this review (minimum nine out of 12 possible points for cross-sectional and ten out of 14 possible points for cohort studies).

**Table 1:** Critical appraisal tool used at this review

Critical appraisal tool
1. Was the research question or objective in this paper clearly stated? (1 point)
2. The study clearly describes exposures and outcomes (1 point)
3. The study clearly describes basic characteristics of participants (1 point)
4. The results were adjusted for possible confounding variables through stratification or multivariate analysis (1 point)
5. Statistical test i. The statistical test used to analyse the data is clearly described and appropriate, and the measurement of the association is presented, including confidence intervals and probability level (1 point) ii. The statistical test is not appropriate, not described or incomplete
6. The study informs the loss to follow-up characteristics: numbers and reasons (1 point—cohort studies only)
7. Participants were followed for the same time or the study was adjusted for different follow-up times (1 point—cohort studies only)
8. The measures used for the main outcomes were accurate: description of the technique for the diagnosis of malaria and antibody measurement (1 point)
9. The demographic characteristics were comparable or adjusted: age and geographic area (1 point)
10. The participants of different groups were recruited in the same period of time (1 point)
11. Representativeness of the sample: i. Truly representative of the average in the target population: all subjects or random sampling (2 points) ii. Somewhat representative of the average in the target population: non-random sampling (1 point) iii. Selected group of users
12. Sample size: i. Justified and satisfactory (1 point) ii. Not justified
13. Ascertainment of the exposure (risk factor) i. Validated measurement tool or non-validated measurement tool, but the tool is available or described (1 point) ii. No description of the measurement tool

Cross-sectional studies score a maximum of 12 points. Cohort studies score a maximum of 14 points

## Results

### Identification and general description of included studies

**Fig. 1:**
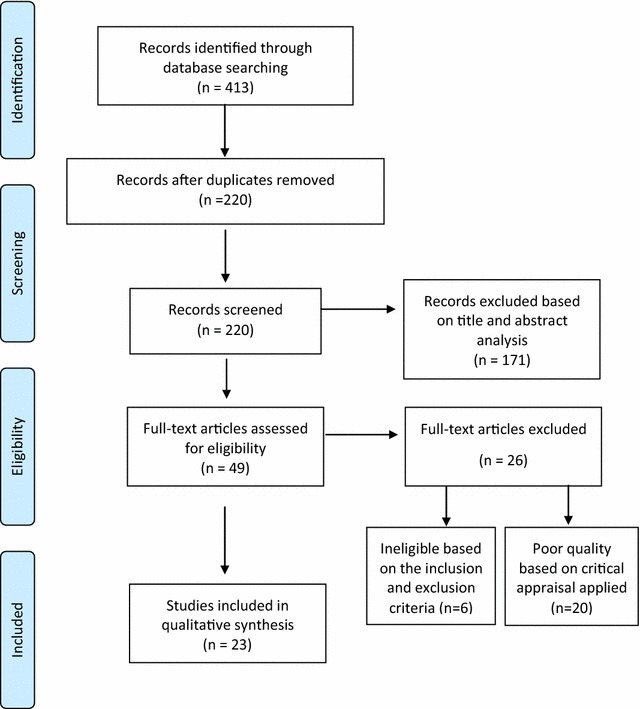
Flow chart with a summary of the articles included in this systematic review. Titles and abstracts were screened from 220 papers. Of these, 171 non-relevant articles were excluded because reported data on imported cases in non-endemic areas included febrile patients attending healthcare facilities, serologically tested convalescent individuals, reported non-human experimental data, were case-reports or presented any other exclusion criteria identifiable in their abstracts. A further six papers were found to be ineligible after reading the 49 full-text articles assessed for eligibility criteria (lacked relevant information on the variables of interest: ascertainment of malaria diagnosis, description of the serological method used, target population and representativeness of general population). Of the 49 potentially eligible papers, 20 were considered to be of poor quality (scored <70% when the critical appraisal tool was applied) and were therefore excluded

The search strategy yielded 413 papers across the three different databases used (Medline, EMBASE, LILACS). After removing duplicated papers, 220 articles were screened. Based on their titles and abstracts, 171 non-relevant papers were excluded and 49 full-text articles were assessed for eligibility. Six of these papers were excluded based on the inclusion and exclusion criteria (lacked relevant information on the variables of interest: ascertainment of malaria diagnosis, description of the serological method used, target population and representativeness of general population). Twenty articles scored <70% when the critical appraisal tool was applied and were therefore excluded. Hence, 23 studies were included in this review for the qualitative synthesis of the published papers (Fig. 1).

The studies were published between 1975 and 2016, with a larger proportion of these after the year 2000. Only five studies were published in the 1990s and a single one in 1975. Details of the 23 articles included in this systematic review are shown in Table 2.

**Table 2:** Summary of seroprevalence studies included in the systematic review by year of publication

Identification (references)	Year of publication	State	Study design	Sample size^a^	Target antigen^bc^	Overall seroprevalence (%)	95% CI
Rodrigues da Silva et al. [34]	2016	Rondônia	Cross-sectional	545	PvMSP9-RIRII	58	53.86–62.14
					PvMSP9-E795/A808	32.5	28.57–36.43
Sanchez-Arcila et al. [30]	2015	Rondônia	Cross-sectional	279	PvMSP1_19_	66	60.44–71.56
					PvAMA-1(*Picchia pastoris*)	63	57.33–68.67
Cunha et al. [28]	2014	Pará	Cross-sectional	1330	PfMSP1_19_	25.6	23.25–27.95
					PfAMA-1	12.7	10.91–14.49
					PvMSP1_19_	36.3	33.72–38.88
					PvAMA-1	32.5	29.98–35.02
Ferreira et al. [38]	2014	Rondônia	Cross-sectional	253	PvRMC-RBP1	47.1	40.95–53.25
					PvRBP1-_23-751_	60	53.96–66.04
Versiani et al. [41]	2013	Amazonas	Cohort	308	PvMSP1 (N-terminus)	35.4	30.06–40.74
Lima Junior et al. [33]	2012	Rondônia	Cross-sectional	276	PvMSP1_19_	86.7	82.69–90.71
					PvMSP3-alpha	77	72.04–81.96
					PvMSP9	76	70.96–81.04
Kano et al. [29]	2012	Amazonas	Cross-sectional	432	PvDBP	49.5	44.79–54.21
					PvMSP1_19_	86.7	83.5–89.9
Lima Junior et al. [32]	2011	Rondônia	Cross-sectional	282	PvMSP3-alpha	78	73.17–82.83
Souza-Silva et al. [40]	2010	Acre	Cohort	366	PvDBP	18.6	14.61–22.59
Lima Junior et al. [31]	2008	Rondônia	Cross-sectional	306	PvMSP9	74	69.1–78.91
Ladeia-Andrade et al. [27]	2007	Amazonas	Cross-sectional	812 778	PvMSP1_19_	66.3^d^	62.93–69.43
					PfMSP1_19_ (*Saccharomyces cerevisae*)	53.5^d^	49.96–56.95
Bastos et al. [18]	2007	Acre	Cohort	376	PvMSP1_19_	54.3	46.29–59.34
Suarez-Mutis et al. [24]	2007	Amazonas	Cross-sectional	98	PvMSP1_19_	46.9	37.02–56.78
Scopel et al. [25]	2007	Acre	Cohort	356	PfMSP-2 S20	28.1	23.43–32.77
					PfMSP-2 FC27	22.2	17.88–26.52
					PfMSP-2 AM89	7.3	4.6–10
					PfMSP-2 3D7	6.2	3.69–8.71
					PfMSP-2 FUP/CP	6.2	3.69–8.71
Nogueira et al. [39]	2006	Rondônia	Cohort	429	N-terminus–PvMSP1	35^d^	30.6–39.59
					PvMSP1_19_	41.5^d^	36.93–46.21
Tran et al. [37]	2005	Rondônia	Cross-sectional	294	PvDBP	67	61.63–72,37
					PvRBP-1	66	60.59–71.47
Marcano et al. [15]	2004	Roraima	Cross-sectional	101	Pf blood stages (IFAT)	72.1	63.35–80.85
Alexandre et al. [26]	1997	Amazonas	Cross-sectional	241	PfR44	26.5^e^	20.93–32.07
					PfAg-T	57.7^e^	51.43–63.91
Arruda et al. [14]	1996	Pará	Cross-sectional	430	PfCS	44^f^	39.33–48.71
					Pf blood stage (IFAT)	68^f^	63.54–72.36
					PvCS	60.1^e^	55.42–64.68
					Pv blood stage (IFAT)	73.2^e^	68.98–77.36
Ferreira et al. [17]	1994	Rondônia	Cohort	50	Pf Exoantigens	30^g^	17.3–42.7
					Pf Somatic Antigens	40^g^	26.42–53.58
Oliveira-Ferreira et al. [16]	1992	Rondônia	Cross-sectional	1617	PfCS (Immunoradiometric)	5.9	4.75–7.05
					Pf Blood stage (IFAT)	49.2	46.76–51.64
Kremsner et al. [23]	1992	Acre	Cross-sectional	645	PvCS	17.3^f^	14.33–20.17
					PvCS (VK247)	4.8^f^	3.11–6.39
Jeffery et al. [19]	1975	Mato Grosso	Cross-sectional	4270	Pf and Pv blood stage (IFAT)	10.5^h^	9.58–11.42

^a^Sample size refers to number of participants for whom serology was determined

^b^If other serological methods were used instead ELISA, it is shown in parenthesis

^c^If other expression systems were used to produce recombinant antigens instead *E.coli* in fusion with histydine tag (His6) or Glutationa S-transferase (GST), it is shown in parenthesis

^d^Combined cross-sectional surveys

^e^Average seroprevalence in all age groups

^f^Average seroprevalence for all cross-sectional surveys

^g^Results shown for the last survey conducted

^h^Average prevalence for both antigens (*P. falciparum* and *P. vivax* blood stages) across four surveys

The majority of studies (14 out 23) used microscopic observations of thick blood smears to determine malaria infection, whereas others used PCR in addition to microscopy.

The studies selected were conducted among populations living in six different states within the Brazilian Amazon Basin (Rondônia, Amazonas, Acre, Pará, Mato Grosso, Roraima). Most of them analysed humoral immune responses among distinct populations living in Rondônia State. Two different studies were conducted among indigenous populations. One of them took place in four isolated indigenous tribes from the Amazon region [14] and the other one in Roraima among the Yanomami Amerindians [15].

Five studies reported serology results on *P. falciparum*, 14 papers assessed *P. vivax* and four articles reported results on both *Plasmodium* species. The majority of published papers were cross-sectional surveys (82.6%) and only four cohort studies were included in this review.

Enzyme-linked immunosorbent assay (ELISA) alone or in combination with other methods, was the chosen serological assay used in 21 (91.3%) of the included papers. Indirect fluorescent antibody test (IFAT) was used in five studies published between 1975 and 2004. Furthermore, a considerable number of *P. falciparum* and *P. vivax* antigens were used to determine IgG-specific antibody responses among different populations in the Brazilian Amazon endemic region (Table 2).

The 23 retrieved sero-epidemiological studies investigated the IgG immune responses to several malaria vaccine candidates. The majority of them included recombinant proteins, which represent the merozoite surface proteins (MSPs) of both *P. vivax* and *P. falciparum*. Other recombinant antigens have also been included, such as CS, AMA1 and DBP as well as *P. falciparum* and *P. vivax* blood stages crude extract (Table 2).

It is worth noting that among the 23 selected studies, eight also examined IgG subclasses responses to malarial antigens. Only four studies examined the profile of IgM responses in parallel to IgG antibodies against *P. falciparum* whole parasites antigens [15–17] or against recombinant variant-specific PvMSP1 antigens [18].

### Studies on seroprevalence reflect changes in temporal malaria distribution in the Brazilian Amazon Basin

The species-specific antigens used in the eligible studies were consistent with the case distribution data for *P. falciparum* and *P. vivax* reported by the Brazilian Ministry of Health since 1960. The first paper on malaria sero-epidemiology included in this review was published in 1975 by Jeffery et al. [19] representing pioneer work in Brazil, pointing out the usefulness of a serological method for the characterization of malaria endemicity. This repeated cross-sectional survey studied 4270 individuals, males and females and both children and adults living in seven different communities in Mato Grosso State (MT) and six communities in what is nowadays known as the Mato Grosso do Sul State (MS). The authors sampled members of urban and rural communities from April 1970 to September 1971 to account for variations in malaria transmission patterns at the rainy or dry seasons. At that time, similar proportions of *P. falciparum* and *P. vivax* confirmed cases were diagnosed in the Brazilian endemic region. The authors ascertained malarial infection by thick blood smears and applied IFAT based on sera eluted from paper filter in populations where constant movement of people and variations in transmission intensity made the collection of reliable malariometric information particularly difficult. The overall seroprevalence (both *P. falciparum* and *P. vivax*) varied from 4.3 to 13.6% with a prevalence of malaria infection ranging from 0.8 to 2.3% across four surveys. Stratified seroprevalence per age group is reported only for the Cuiaba region (MT), where 2.9–7.4% of children aged 5–14 years had positive antibodies against *P. falciparum* or *P. vivax*. Of note is the fact that based on such extremely low parasite rates and relatively low IFAT positivity, the large-scale insecticide-spraying programme in Mato Grosso State was interrupted during the 1970s.

During the 1980s, malaria cases increased in the Brazilian Amazon region as an effect of environmental and demographic changes during the military dictatorship regime [2, 20, 21]. Despite that decade being considered crucial in shaping the new malaria epidemiological scenario in the Brazilian Amazon region, none of the sero-epidemiological studies published in the 1980s could be selected for this systematic review. There was one potential study conducted in 1982 among the 49 full-text articles assessed for eligibility written by Ferraroni et al. [22]. However, this survey did not provide enough information regarding infection rates among the studied population and was excluded from the review.

The five selected works carried out during the 1990s followed the malaria distribution trend in the late 1980s when similar number of malaria cases due to *P. falciparum* and *P. vivax* were registered in the Brazilian Basin. Indeed, two out of five of those studies, which were conducted in Acre and Pará States, used *P. falciparum* and *P. vivax* total proteins as target antigens to detect specific antibodies among endemic populations [14, 23]. Two studies used *P. falciparum* blood-stage antigens as well the repeated epitopes of circumsporozoite protein [14, 16].

The majority of papers published during the 2000s focused on *P. vivax* antigens (MSP1, MSP3, MSP9, AMA1, RBP1, DBP) (Table 2) reflecting this species predominance in the Brazilian endemic regions after implementation of the Programme for the Intensification of Malaria Control in October 1999 and the National Programme for Malaria Prevention and Control in 2005. In fact, only three studies aiming to evaluate the antibody responses to *P. falciparum* antigens were conducted during the 2000s [15, 24, 25].

### Antibody responses to *Plasmodium falciparum* and *Plasmodium vivax* CS antigens and their association with malaria exposure

Few cross-sectional studies aimed to characterize the antibody responses against (CS) proteins of *P. falciparum* and *P. vivax*. The three selected studies were conducted during the 1980s in three different states (Acre, Rondônia, Pará) depicting low transmission rates (parasite prevalence <10%) [14, 16, 23]. Interestingly, these heterogeneous studies revealed an association between anti-CS antibodies and age as detailed below. The first study used synthetic peptides representing the repeat region of the CS proteins for *P. vivax* (classic and VK247 variant) and *P. falciparum* [23]. Two surveys were conducted in the state of Acre involving 645 individuals living in two different communities (about 10% of total population). Acute malaria infection varied from 1.2 to 5.3% for *P. vivax* and from 0.625 to 3.3% to *P falciparum* at the surveyed communities. While the seroprevalence of anti-CS targeting classic *P. vivax* varied from 9 to 25%, the VK247 variant was less frequently observed varying from 1.2 to 10% among sampled subjects. The prevalence of antibodies to both *P. falciparum* and *P. vivax* CS proteins was higher in areas of recent migration mainly in 1987. Furthermore, an overall decline in the seroprevalence of anti-CS antibodies in 1990 compared to 1987 was reported and may reflect a significant decrease in the number of malaria cases reported by the Brazilian Ministry of Health at that time as a consequence of DDT spraying. Moreover, antibodies against CS proteins of both *P. falciparum* and *P. vivax* increased with age (p < 0.001) in the 1987 and 1990 surveys. Taken together, these results point out an association between anti-sporozoite antibodies and malaria exposure.

A similar association was described in a study comprising five cross-sectional surveys between 1986 and 1989 in four different communities in Rondônia State [16]. A total of 1617 people were included in this study, which assessed anti-*P. falciparum* CS antibodies. A seroprevalence of 6% was revealed but no relationship observed between the frequency of anti-sporozoites (CS) and gender, time of residence or malaria infection. However, a significantly higher frequency was observed in individuals over 30 years old (p < 0.001) and in those with a history of more than nine previous malaria episodes (p < 0.001).

In agreement with the two previous published papers, data collected from cross-sectional surveys conducted between 1986 and 1988 among four indigenous Brazilian tribes in Pará State (n = 430 natives) revealed an increased anti-sporozoite seropositivity according to age for both *P. vivax* and *P. falciparum* [14].

### Cross-sectional studies related to *Plasmodium falciparum* blood-stage antigens

Six cross-sectional studies evaluated the IgG profiles to total *P. falciparum* asexual blood-stage antigens [14–16, 19, 24, 26] in inhabitants of different localities within the Brazilian Amazon. The areas studied are considered hypo/meso-endemic with variable and unstable malaria transmission. Two different techniques, IFAT and ELISA, were used to measure anti-total blood stage IgG across the populations studied. The relationship between seroprevalence and cumulative exposure to malaria was heterogeneous among the studies: some studies evidenced an increase in seroprevalence with age [19, 24, 26] while others suggested no association between the proportion of responders and age of sampled subjects [14–16].

Recombinant antigens of *P. falciparum* were also used in cross-sectional studies aiming to define the patterns of naturally occurring antibodies in different epidemiological settings within the Amazon Basin [26–28]. Such studies used recombinant proteins representing three different antigens, which have been considered potential antigen candidates for vaccines against falciparum malaria: the 72 kDa heat-shock protein from *P. falciparum* (Pf72/Hsp70-1), the 19 kDa fragment of PfMSP-1 that remains on the surface of merozoite during invasion (PfMSP-1_19_) and the apical membrane antigen 1 of 3D7 strain (PfAMA1).

A cross-sectional survey conducted in Candeias do Jamari (Rondônia State), a small town in a hypo-endemic area with unstable malaria transmission, selected 241 non-immune migrants using household-based randomization criteria [26]. Blood stage antibodies against the Pf72/Hsp 70-1 protein were measured by ELISA and stratified by age group. Both prevalence and levels of specific antibodies were associated with age and time of residence in an endemic area. Only 6.6% of participants had a positive blood film for malaria and they were all symptomatic (56% of these due to *P. falciparum*).

Two different studies measured naturally acquired IgG antibodies to the conserved 19-kDa C-terminal region of the merozoite surface protein 1- PfMSP-1_19_. Two cross-sectional surveys enrolling 594 individuals (both children and adults) living in riverine communities along the Jau and Unini rivers (Amazonas State) evidenced a seroprevalence around 52%, which was positively correlated with age (OR 1.04 95% CI 1.019–1.06 p < 0.0001) [27]. Another sero-epidemiological study enrolled 1330 inhabitants of six different municipalities in Para State (urban, rural, migrant, native populations) between 2006 and 2010 [28]. Despite the low parasite prevalence at those six localities (ranging from 0 to 5%), a considerable overall serological response to PfMSP-1_19_ antigen (25.6%) was reported. This latter study also evaluated the humoral immune response to the apical membrane antigen 1 (AMA1) of *P. falciparum.* An overall prevalence of 12.7% was detected, probably reflecting the little contribution of *P. falciparum* to the malaria burden at the different localities studied during the years of 2006–2010.

### Cross-sectional studies and antibody responses to *Plasmodium vivax* antigens

A total of 13 cross-sectional studies investigated humoral responses to *P. vivax* antigens. Among them, two works reported IgG responses to total *P. vivax* asexual blood stage antigens measured by IFAT [14, 19]. As already mentioned above in relation to *P. falciparum*, the relationship between seroprevalence and age was contrasting, based on data from those two papers.

Most sero-epidemiological studies in the Brazilian Amazon Basin has been focused on the PvMSP-1_19_ antigen. Among 13 cross-sectional studies, five were conducted aiming to measure the naturally acquired antibodies to such conserved fragment of *P. vivax* merozoite. Different papers have reported a positive association between anti-PvMSP-1_19_ antibodies and malaria exposure as detailed below.

Suarez-Mutis et al. [24] sampled 98 individuals over 2 years, living in five small riverine communities near Barcelos (Amazonas State). The study was designed to evaluate the continuous malaria transmission in the area and the possible role played by asymptomatic carriers. Only 1.02% of inhabitants had a malaria-positive blood film but 20.4% cases of *P. vivax* infection were detected by PCR. A seroprevalence of 46.9% for PvMSP-1_19_ was reported and the probability of a positive IgG specific response was 3.44 times higher in individuals older than 15 years in comparison to those younger. Similar results were described by Ladeia-Andrade et al. [27] across two sectional surveys conducted among 594 endemic residents (both children and adults) from riverine communities along the Jau and Unini rivers (Amazonas State). Anti-PvMSP-1_19_ was positive in 69.6%, half way through the rainy season, and 64.0% at the middle of the dry season. Age was positively correlated with seropositivity (OR 1.04 95% CI 1.019–1.06 p < 0.0001). Furthermore, very strong evidence for an association between the number of previous malaria episodes and anti-PvMSP-1_19_ antibodies was observed among 432 subjects living in Rio Pardo, Amazonas State, in a cross-sectional study conducted in 2008 [29].

A recent study demonstrated that serology could be used to measure and monitor transmission of malaria in the Brazilian Amazon endemic region [28]. The authors determined the antibody levels and seroconversion rates to *P. vivax* merozoite antigens (PvMSP-1_19_ and PvAMA1) in individuals living in areas of varying *P. vivax* endemicity in Pará State. Seroprevalence for any *P. vivax* antigens ranged from 19.6% (low transmission) to 77.5% (high transmission) corroborating the official Brazilian annual parasite index (API). However, low parasite prevalence (0–5%) was detected in participants from those six localities.

Data on antibodies to PvMSP-1_19_ and PvAMA1 was also extracted from a paper published by Sanchez-Arcila et al. [30] that evaluated the influence of intestinal parasites in *P. vivax*-specific responses. A total of 279 people (14–43 years old) from a rural settlement were included. Overall seroprevalence was 74% for any antigen, 66% for PvMSP-1_19_ and 63% for PvAMA-1. Interestingly, responses to both PvAMA1 and PvMSP-1_19_ were lower in individuals with intestinal parasites.

Other MSPs antigens have also been evaluated among Amazonian populations. Three papers included in this review used different target antigens for assessing seroprevalence of *P. vivax* in communities in Rondônia State, enrolling both migrant and native populations. The first paper published in 2008 [31] analysed 306 samples using the PvMSP-9 antigen (variants RIRII, RII and NT). An overall seroprevalence of 74% was found with 13% of blood films being positive for malaria. A positive correlation between years of residence in the area and IgG antibody reactivity index was found for all variants in both communities (highest *p* value = 0.02). The second paper by the same group [32] used PvMSP-3 variants as the target antigen in 282 samples. An overall seroprevalence of 78% (for any positive variant) was found with a 12% prevalence of infection. Seropositivity correlated positively with years of residence in an endemic area (p < 0.05) and was also associated with the number of previous malaria episodes. The third published paper [33] sampled 276 individuals and the target antigens used were PvMSP1_19_, PvMSP3-α and PvMSP-9. The frequency of responders shows that IgG antibody reactivity to PvMSP-1_19_ was 86.7% and the frequency of positive individuals for at least one of the recombinant proteins representing PvMSP-3a and PvMSP-9 sequences was 77 and 76%, respectively. Another paper conducted at those same localities in Rondônia State verified 58% of positive antibody responses to recombinant PvMSP9-RIRII and 32.5% to PvMSP9 E795-A808 protein [34]. In agreement to previous studies, seropositive individuals for PvMSP9-RIRII had a higher median of years of residence in the endemic area.

The vivax Duffy binding protein (PvDBP), a leading malaria vaccine candidate that plays a critical role in *P. vivax* erythrocyte invasion [35, 36], has been used in two different sero-epidemiological studies. Tran et al. [37] assessed the seroprevalence of naturally acquired PvDBP-RII in three communities in the state of Rondônia. Native and migrant populations were evaluated in this paper, which included 294 individuals (children and adults). An overall seroprevalence of 67% was found for anti-PvDBP. The prevalence of acute malaria infection varied from 8 to 37% across the three communities. The authors found weak evidence for an association between seropositivity and increasing age and time of residence in the endemic area (OR 2.25 and 95% CI 0.79–6.43). Evidence was stronger for individuals living in the endemic area for more than 30 years (OR 3.3 and 95% CI 1.17–9.35) and a higher number of previous malaria episodes. Individuals with more than 15 previous malaria episodes had 6.98 times the odds of being anti-PvDBP positive (95% CI 1.79–27.17) than individuals with no previous malaria episodes. The PvDBP was also evaluated in a well-consolidated agricultural settlement of the Brazilian Amazon region [29] showing similar results. The frequency of antibodies against PvDBPII–IV was 49.5% and age was the only predictor significantly associated with the presence of anti-PvDBP antibodies (adjusted OR 1.05, 95% CI 1.02–1.08, p = 0.005). Each additional year of age increased the probability of having anti-PvDBP antibodies by 5%.

Reticulocyte binding protein 1 (PvRBP1), another protein involved in the reticulocyte-specificity of *P. vivax* blood-stage infections was investigated in two published papers. The first paper measures specific IgG antibodies to five recombinant fragments spanning the length of the extracellular domain of PvRBP1 [37]. Seroprevalence for any of the five PvRBP1 fragments was 66%. Positive IgG responses were correlated to the level of malaria exposure (past infections and years of residence in the endemic area) after adjustment for age. Recently, Ferreira and collaborators [38] used a chimeric synthetic peptide and the non-chimeric recombinant reticulocyte binding protein to assess the seroprevalence in a cross-sectional study, which included 253 samples. Total IgG responses were observed in 47.1% of the population to the chimeric PvRMC-RBP1 and in 60% to the non-chimeric protein PvRBP123-751. There was a significant difference in the response to these two recombinant proteins (p = 0.0031). Past malaria infections and time of residence in endemic area were associated with higher levels of IgG against both antigens. No association was found between seropositivity and time since last malaria episode.

### Cohort studies

Only six cohort studies were included in this systematic review. These longitudinal studies focused on different antigens of *P. falciparum* [17, 25] and *P. vivax* [18, 39–41]. A crucial point to be mentioned here is that these six published papers used different periods of follow-up after parasite detection to determine asymptomatic infections.

Regarding *P. falciparum*, two studies were conducted in two distinct epidemiological settings. The first cohort study included in this review was published in 1994 by Ferreira and collaborators [17]. The authors followed 50 individuals aged one-51 years for 1 year between 1991 and 1992. Participants lived in a rural settlement (total population of 180) in Urupá, Rondônia State, a hypo-endemic area for malaria at that time. Most of the participants were migrants from non-endemic areas of the country. An outbreak took place during follow-up with 48 malaria attacks (28 due to *P. falciparum*) among 25 individuals. Seroprevalence for *P. falciparum* somatic and exo-antigens increased after the outbreak. An increase from 13 to 30% IgG positivity for exo-antigens and from 20 to 40% for somatic antigens was reported. No association between previous malaria immunity and the profile of malaria response could be drawn from this study.

The other cohort study analysed the prevalence of antibodies recognizing variant antigens representing the MSP-2 of *P. falciparum* (FC27 and 3D7 dimorphic allelic families) among 356 individuals living in a rural community in Acre State [25]. Seropositivity for five MSP-2 variant antigens ranged from 6.2 to 28.1%, the S20 variant being the most recognized. Evidence for a correlation between the level of IgG antibodies and age and time of residence in the Amazon was observed. Cumulative malaria exposure or the presence of variant-specific antibodies matching the MSP-2 type in infecting parasites were not considered major predictors of clinical severity.

With regard to *P. vivax*, it is worth mentioning that combined results from two studies showed an evidence of association of PvMSP1 N-terminal responses and time of residence in the Brazilian endemic regions [39, 41]. Nogueira et al. [39] conducted three cross-sectional surveys between September 1998 and September 1999 among 180 individuals living in a riverine community in Rondônia State. A positive association between time of residence in the endemic area and seropositivity was found for individuals living in the community for more than 15 years (OR 30.35 95% CI 2.42–381 for individuals living in the community for more than 30 years). A small cohort was followed up for 1 year and a hazard risk for acquiring malaria of 2.95 (95% CI 1.14–7.65) for individuals with negative serology (N-terminus PvMSP-1) was described.

A second paper from the same research group analysed the profile of antibodies against the N-terminal region of the PvMSP-1 in a 1-year follow-up conducted among 308 individuals living in a rural community in Amazonas State [41]. Seropositivity of 35.4% for the target antigen and 14% parasite infection prevalence were reported. Age was associated with seropositivity (OR 1.02 95% CI 1.01–1.03, p = 0.002; a 2% increase for each additional year in age). This study also analysed the relationship between asymptomatic infection and the presence of such specific antibodies. Individuals with asymptomatic malaria (detected by PCR only) had an OR of positive antibody of 2.45 (95% CI 1.06–5.69, p = 0.036) compared with malaria negative individuals. A Kaplan–Meier time to event analysis was performed with 293 participants. The total IgG anti-MSP1 (N-terminus) was detected to be an important protective factor against vivax malaria clinical attacks (p = 0.029).

Another cohort study included in this review was published by Bastos et al. [18] in 2007. People living in a small community in Acre were followed up for 15 months where 376 subjects were considered eligible for the serological study. Over 60% of the study population were migrants. Variants of PvMSP1 were used as target antigen and 54.3% had a positive serology (ELISA) for PvMSP1_19_. The authors found a positive correlation (p < 0.005) between IgG PvMSP-1 antibodies and time of residence in the Amazon.

The prevalence of anti-DBP antibodies was investigated in 366 people living in Ramal do Granada (Acre State) [40]. Two cross-sectional surveys were conducted but only the baseline data are analysed in this review. Anti-DBP antibodies were present in 18.6% of individuals. Time (years) of residence in the Amazon region was associated with the presence of IgG antibodies (OR 1.02 95% CI 1.0–1.04 representing a 2% increase in the odds of having a positive anti-DBP antibody for every 1-year increase in residence). Age, past or current malaria infection were not considered strong predictors of antibody positivity.

## Discussion

This systematic literature review included 23 studies reporting malaria seroprevalence data in the Brazilian Amazon endemic region. Most of the studies (60.87%) reported data on *P. vivax* exclusively, reflecting the epidemiological changes in *Plasmodium* species distribution in Brazil. Malaria cases shifted from predominantly caused by *P. falciparum* in the 1960s to *P. vivax*, the latter now being responsible for over 80% of cases in the country. Studies included in this review were highly heterogeneous and reported seroprevalence varied with the transmission risk in the area, target antigen used and target population (migrant, indigenous, native or mixed communities).

Since the Global Malaria Eradication Programme proposed by the WHO in 1955, serology has been used to measure malaria exposure and was a prominent method in early elimination attempts reported in papers published in the 1970s [19, 42–44]. As these elimination attempts were abandoned, the use of serological characterization was discouraged and was no longer found on subsequent publications. With little use over several decades, these serologic assays lacked standardized, reproducible and objective methods [42, 45]. Recent technological improvements mean that serology has now become a much more robust tool for transmission measurement, but there is still a need to standardize ELISA protocols and antigens [11]. This scenario is reflected in this systematic review, which highlights the significantly more frequent use of serological methods in malaria over the past decades, as depicted from the progressively increasing number of papers published since 1975.

The Brazilian Amazon region is characterized by highly variable and unstable malaria transmission. The risk of acquiring malaria varies substantially even within the same state. These different transmission patterns could explain some of the variation in seroprevalence found in the selected studies, in addition to the sensitivity of the chosen target antigen. Twelve different antigens were used in *P. vivax* studies and another ten different targets in *P. falciparum* studies. Thus, a gross seroprevalence comparison across these communities with variable malaria transmission using several different targets would be inappropriate.

It is important to note that studies involving native indigenous populations are under-represented. Only two papers [14, 15] evaluated indigenous communities in Pará and Rondônia States. However, genetic indigenous backgrounds, epidemiological settings, period of evaluation, and antigens tested were markedly distinct between the studies preventing any comparative analysis. There are currently no updated malaria sero-epidemiological surveys among the indigenous communities in the Brazilian Amazon. A better understanding of malaria transmission in these vulnerable communities is urgently needed. Indeed, the first National Survey of Indigenous People’s Health and Nutrition in Brazil conducted between 2008 and 2009 gives insufficient information on malaria diagnosis and outcomes among indigenous children and women [46].

### Target antigens and association with malaria exposure

There seems to be suggestive evidence that higher titres of IgG antibodies against some antigen targets (but not all) correlate with the number of years living in the endemic area and not necessarily with age. Sero-epidemiological studies conducted in Africa have shown an association with the presence of malaria antibodies and age, which has been used as surrogate marker for malaria exposure [47, 48]. Here it is worth considering the particular epidemiological scenario of malaria in the Brazilian Amazon Basin. In the late 1960s, a Brazilian national demographic policy stimulated migration from the non-endemic southern areas towards the Amazon in order to increase population density in the underpopulated northern areas of the country. These previous migration policies translate into a higher proportion of migrant population with less long-term exposure to malaria living in the communities assessed by the studies reported here. Thus, a clear correlation between age and malaria exposure was not expected in the majority of the selected sero-epidemiological studies, especially those conducted during the 1990s. However, even for studies enrolling inhabitants from stable communities, such as agricultural settlements and riverine populations, there was not a strong association between age and the profile of specific antibody responses. Living in the endemic area for more than ten to 15 years seems to be better correlated with seropositivity for malaria antigens based on the selected studies.

Three selected heterogeneous studies conducted during the 1980s [14, 16, 23] evidenced an association between anti-CS antibodies and age and therefore seem to reflect the cumulative exposure to malaria-infected mosquitoes. Interestingly, the antibodies against synthetic peptides containing the tandem repeated immunodominant epitope of *P. vivax* [16] and *P. falciparum* [16, 23] CS proteins were associated to age even in migrant populations with limited exposure to malaria. Similar association between sporozoite-specific antibodies and stratified age was found in indigenous tribes [23]. Considering that studies enrolling the CS proteins have not been carried out since the early 1990s in endemic Brazilian populations, an interpretation of the potential use of specific antibodies as a biomarker of malaria exposure should be cautiously analysed.

Few studies attempted to investigate the association between seropositivity and months since the last malaria episode. However, the cross-sectional design adopted by most of the authors is subject to recall bias from individuals surveyed. The criteria used to ascertain previous malaria infections is not clearly defined in any of the reported cross-sectional studies, apart from the paper assessing PvCS and PfCS antigens where positivity against these targets was clearly associated with individuals reporting around ten previous malaria episodes [16].

The 19 kDa conserved fragment of MSP-1 from both *P. falciparum* and *P. vivax* has been considered a potential marker for protection against malaria as well as a surrogate marker for malaria exposure in geographically diverse populations [49, 50]. This systematic review corroborates those findings describing an association between PvMSP-1_19_/PfMSP-1_19_ and malaria exposure parameters (age, time of residence in endemic areas, and number of previous malaria episodes) in cross-sectional sero-epidemiological studies [24, 27–29]. Although the gene sequences of MSP-1_19_ antigens are exclusive to each *Plasmodium* species, considerable homology has been reported among them [51] and, therefore, cross-reactivity in co-endemic areas could be expected. However, a recent study demonstrated that IgG antibodies to recombinant MSP-1_19_ antigens of the four major human malaria parasites are species-specific in Zimbabwean populations [52]. Here none of the selected studies assessed the inter-species cross-reactivity of MSP-1_19_ serological responses, an issue that remains to be established among Brazilian Amazonian populations.

It has been suggested that serological methods might be used to distinguish between relapse and new *P. vivax* infections by measuring exposure to mosquito saliva through detection of anti-saliva antibodies or through the measurement of hypnozoite-specific signals [42]. However, none of the studies included in review identified target antigens or described differences in antibody levels in relation to relapses or new infections. Relapse infections were not distinguished from new infections in any of the reported studies. One important point to be addressed is the latest sequencing data suggesting that *P. vivax* has a more variable and diverse genome than *P. falciparum*. Thus, multi-antigenic approaches such as micro-array would allow more detailed screening of the different antigen variants that could possibly be used to distinguish relapses from new infections [53, 54].

### Immunity and asymptomatic infection

Antibodies are important mediators of naturally acquired immunity (NAI) that controls blood-stage parasitaemia, thereby reducing clinical symptoms and life-threatening complications. The role of specific antibodies is evidenced by experimental animal models and, most importantly, classical passive transfer studies in which antibodies from malaria immune adults were successfully used to treat children with severe malaria [55, 56]. Moreover, several studies have evidenced the role of antibodies in NAI, involving protection from infection and severe clinical symptoms. NAI has been extensively demonstrated for *P. falciparum*, and more recently for *P. vivax,* in regions of high and low transmission [57]. Interestingly, NAI is acquired more rapidly in *P. vivax* infection compared to *P. falciparum*, which may be due to the differing biological features related to the two species, such as the ability of *P. vivax* to maintain hypnozoites in the liver. Thus, a differential contribution of antibodies to NAI in *P. vivax* and *P. falciparum* infections is expected. Brazilian malaria distribution since the early 1960s and the fluctuations in proportions of slide-confirmed *P. falciparum* and *P. vivax* infections diagnosed over the last decades represent an ideal scenario to address this puzzling question. Notwithstanding, little is known about NAI in areas of less intense malaria transmission in the Amazon Basin mainly due to few cohort studies being conducted in the region.

Asymptomatic malaria has been a subject of increasing interest in the Brazilian context but is still lacking substantial data. Serology has the potential to detect not only ongoing blood-stage infections missed by conventional microscopy, but also recent infections, either symptomatic or not. Despite this potential, the use of serology for targeting asymptomatic carriers has been rarely reported in Brazil and the available data do not provide conclusive or comparative results.

Sensitive diagnostic methods, such as PCR, and an appropriate follow-up period are required to identify asymptomatic malaria infection. Certainly, these requisites impose substantial costs on epidemiological surveys, which may explain the scarce number of longitudinal sero-epidemiological studies in Brazil. From all the studies that attempted to investigate the association between seropositivity and asymptomatic infection, two cohort studies suggested that PvMSP-1 N-terminal region might be a target for protective immunity [39, 41]. These longitudinal studies used clearly defined criteria to establish the occurrence of asymptomatic infection among well-defined populations in Rondônia and Amazonas States. In fact, these studies prospectively assessed the relationship between seropositivity and time elapsed until next malaria episode and found a protective association for PvMSP-1 N-terminus after a 1-year follow-up. However, such association requires further investigation in other epidemiological settings to validate the N-terminal of PvMSP-1 as a putative protective biomarker in endemic populations.

## Conclusions

Although serology has been recognized as a valid surveillance tool, the vast majority of studies in this review used it as the means to identify potential targets for the development of a vaccine against the disease. Major issues with the representativeness of the sample arise from this approach, as most of the authors used a convenient sampling method instead of structured sample size calculations and random selection of individuals. Despite the bias introduced by the sampling methods used and the focus on identifiable targets for a candidate vaccine, valuable malaria transmission information can still be retrieved from these studies.

The review also highlights the need for longitudinal studies to better understand the role of these antibodies in malaria protection and asymptomatic *P. vivax* infections. There is a gap in research to be filled by better designed, cross-sectional, epidemiological surveys with improved sampling procedures. Surveys focusing on the broader epidemiological and sero-surveillance aspects of malaria seroprevalence are needed, considering the aim of most published studies included in this review was to identify prospective malaria vaccine candidates.

This systematic review reveals that IgG antibodies against two parasite proteins, the CS and the MSP1_19_ proteins, of both *P falciparum* and *P. vivax,* may be considered as putative biomarkers of malaria exposure in areas with variable and unstable malaria transmission in the Brazilian Amazon. However, standardization of target antigens and their relationship with malaria exposure are issues that require extra attention and need to be addressed before seroprevalence methods can be incorporated as a surveillance tool into the Brazilian malaria programme. A useful biomarker to differentiate relapses from new *P. vivax* infections is yet to be explored by further research. Furthermore, the proposed critical-appraisal, score-based, weighted tool developed and used in this study to assess cross-sectional studies still requires further validation.

In conclusion, efforts will need to be made to ensure greater consistence among further sero-epidemiological studies in the Brazilian Amazon endemic region to improve the ability to compare different results that should contribute to understanding malaria transmission.

## Abbreviations

AMA: apical membrane antigen
API: annual parasite index
CI: confidence interval
CS: circumsporozoite
DBP: duffy binding protein
EIR: entomological infection rate
ELISA: enzyme-linked immunosorbent assay
IFAT: indirect fluorescent antibody test
IgG: immunoglobulin G
MSP: merozoite surface protein
NAI: naturally acquired immunity
NOS: Newcastle-Ottawa scale
OR: odds ratio
PAHO: Pan American Health Organization
PCR: polymerase chain reaction
RBP: reticulocyte binding protein
WHO: World Health Organization
